# Draft Genome Sequence of *Bacillus licheniformis* CG-B52, a Highly Virulent Bacterium of Pacific White Shrimp (*Litopenaeus vannamei*), Isolated from a Colombian Caribbean Aquaculture Outbreak

**DOI:** 10.1128/genomeA.00321-16

**Published:** 2016-05-12

**Authors:** Eric J. C. Gálvez, Katerine Carrillo-Castro, Lina Zárate, Linda Güiza, Dietmar H. Pieper, Erika García-Bonilla, Marcela Salazar, Howard Junca

**Affiliations:** a Corporación CorpoGen, Bogotá, Colombia; b Corporación Centro de Investigación de la Acuicultura en Colombia, CENIACUA, Bogotá, Colombia; c Microbial Interactions and Processes Research Group, Helmholtz Centre for Infection Research, Braunschweig, Germany; d RG Microbial Ecology: Metabolism, Genomics & Evolution, Div Ecogenomics & Holobionts, Microbiomas Foundation, Chía, Colombia

## Abstract

*Bacillus licheniformis* strain CG-B52 was isolated as the etiological agent producing a self-limited outbreak of high mortalities in commercial *Litopenaeus vannamei* culture ponds on the Colombian Caribbean coast in 2005. Here, we report its draft genome and three novel extrachromosomal elements that it harbors.

## GENOME ANNOUNCEMENT

Strains belonging to the *Bacillus* genus have been widely used as a probiotic in shrimp and fish aquaculture to improve protection against various pathogens (1–4). A Gram-positive *Bacillus* sp. strain CG-B52 was isolated from hemolymph in diseased *Litopenaeus vannamei* Pacific white shrimp from broodstock raising ponds on the Colombian Caribbean coast. Mortality in these ponds was triggered by stress episodes such as pond transfer, which resulted in outbreak mortalities up to 70%. Shrimp of 0.5 to 12 grams orally exposed to isolate CG-B52 (immersed in ≥10^7^ CFU/ml) developed the lethal disease. After four bacterial culture passages (one every 2 days for 8 days) in R2A media at 30°C, CG-B52 lost its virulent behavior. Sequencing of the initial *Bacillus* sp. isolate CG-B52 was performed to assist in identifying potential pathogenesis determinants. Total DNA was subjected to sequencing on Illumina MiSeq PE 250-bp technology, resulting in a total of 6,291,486 pair-end reads. Quality trimming, *de novo* assembly, and scaffolding were performed using CLC cell assembly version 4.010 (CLC, Denmark). The preliminary assembly consisted of 106 scaffolds, which were used as a reference for the final assembly. The average coverage was 360×, (minimum length, 330 bp; maximum, 506,669 bp; *N*_50_, 217,730 bp). Low coverage contigs (<1×) and contigs shorter than 300 bp were discarded. The final assembly consists of 50 scaffolds, with 3 scaffolds identified as extrachromosomal elements. A complete 16S rRNA gene sequence assembled was used to find the nearest fully sequenced relative as a reference for reorganizing the scaffolds using Mauve version 2.3.1 (5). Genome annotation was performed using RAST server (6) and PGAAP version 2.1 (7).

The final draft genome consists of 4.4 Mb with 45.7% GC content and 4,760 putative coding sequences. Using the SEED (8) functional annotation tool we could not identify any putative toxin or superantigen genes in the genomic-associated contigs. Eighteen genes were classified as invasion and intracellular resistance operons. Comparative genomics revealed that *Bacillus* sp. strain CG-B52 belongs to the *B. licheniformis* species complex and is similar to strain 10-1-A (9). It is the first isolate of that complex reported as having such virulent behavior. We identified the presence of 3 novel extrachromosomal elements: the pB52L contig of 136,597 bp, GC = 35.3% (GenBank no. AVEZ01000043.1), and two plasmids, pB52x of 7,914 bp, GC = 43.1% (GenBank no. AVEZ01000044.1) and pB52y of 6,283 bp, GC = 39.6% (GenBank: AVEZ01000046.1). Their circular nature was experimentally validated by PCR assays, confirming that the contigs’ extremes are a continuous sequence. pB52x has an identity of 66.5% to a reported cryptic plasmid pFL7 from *B. licheniformis* FL7 (10). The low GC contents and absence from other *Bacillus* spp. strains suggested that all plasmids may have been acquired by horizontal gene transfer and are not stably maintained. It could explain the limited nature of the outbreak, which disappeared in less than 4 months. No closely related animal virulence factors or toxins reported in animal pathogens (see, e.g., ref. 11) were detected. The nature of CG-B52’s unusual virulence could be encoded in toxin-antitoxin signatures and hypothetical proteins of unknown functions annotated in its chromosome and episomes.

### Nucleotide sequence accession numbers.

This whole-genome shotgun project has been deposited at DDBJ/EMBL/GenBank under the accession number AVEZ00000000. The version described in this paper is the first version, AVEZ01000000.1.
